# PAR-Induced Harnessing of EZH2 to β-Catenin: Implications for Colorectal Cancer

**DOI:** 10.3390/ijms23158758

**Published:** 2022-08-06

**Authors:** Shoshana Sedley, Jeetendra Kumar Nag, Tatyana Rudina, Rachel Bar-Shavit

**Affiliations:** Sharett Institute of Oncology, Hadassah-Hebrew University Medical Center, Jerusalem 91120, Israel

**Keywords:** GPCR, protease-activated receptors (PARs), EZH2, colon cancer

## Abstract

G-protein-coupled receptors (GPCRs) are involved in a wide array of physiological and disease functions, yet knowledge of their role in colon cancer stem cell maintenance is still lacking. In addition, the molecular mechanisms underlying GPCR-induced post-translational signaling regulation are poorly understood. Here, we find that protease-activated receptor 4 (PAR_4_) unexpectedly acts as a potent oncogene, inducing β-catenin stability and transcriptional activity. Both PAR_4_ and PAR_2_ are able to drive the association of methyltransferase EZH2 with β-catenin, culminating in β-catenin methylation. This methylation on a lysine residue at the N-terminal portion of β-catenin suppresses the ubiquitination of β-catenin, thereby promoting PAR-induced β-catenin stability and transcriptional activity. Indeed, EZH2 is found to be directly correlated with high PAR_4_-driven tumors, and is abundantly expressed in large tumors, whereas very little to almost none is expressed in small tumors. A truncated form of β-catenin, ∆N133β-catenin, devoid of lysine, as well as serine/threonine residues, exhibits low levels of β-catenin and a markedly reduced transcriptional activity following PAR_4_ activation, in contrast to *wt* β-catenin. Our study demonstrates the importance of β-catenin lysine methylation in terms of its sustained expression and function. Taken together, we reveal that PAR-induced post-transcriptional regulation of β-catenin is centrally involved in colon cancer.

## 1. Introduction

Despite the emerging role of G-protein-coupled receptors (GPCRs) in a wide array of physiological and disease functions, knowledge of their regulation of post-translational signaling is still incomplete [1,2,3,4]. Whereas GPCR post-translational modification (PTM) involves phosphorylation on serine/threonine residues by a GPCR receptor kinase family (GRK), ubiquitination for targeted degradation, SUMOylation, S-nitrosylation, tyrosine sulfation, and methylation [5], our knowledge of their signaling induced post-translational involvement is lacking.

GPCRs control many aspects of tumorigenesis, including proliferation, invasion, survival at secondary sites, and several cancer-associated signaling pathways [6]. As a subfamily of GPCRs, Frizzled (FZD) receptors play a pivotal role in development, tissue patterning homeostasis, and cancer. Activation of the Wnt/β-catenin signaling pathway, also called the Wnt canonical pathway, involves the stabilization of β-catenin following ligation of Wnts to FZDs and recruitment of low-density lipoprotein-related protein 5/6 (LRP5/6) coreceptors [7]. Inactivation of β-catenin takes place following its phosphorylation tagging at serine S45 by CK1, followed by S33, S37 and T41 phosphorylation by GSK3β [8]. This phosphorylation cascade labels β-catenin for degradation. β-catenin can then be recognized by the E3 ligase βTrCP, which mediates the ubiquitination of lysine residues K19 and K49, leading to proteasome-dependent degradation [9]. Upon association of Wnts with their receptor and coreceptors, the degradation complex is inhibited. β-catenin is then released, which is translocated to the nucleus, consequently prompting the expression of Wnt-β-catenin target genes downstream [10]. 

It has been well established that the main resistance to standard of care chemotherapy in colorectal cancer (CRC) cannot be explained only on the basis of genetic mutations. Evidence supports the notion that the majority of CRCs possesses colorectal cancer stem cells (CSCs) that drive tumor growth and play a central role in chemoresistance. CSCs have been identified on the basis of nuclear β-catenin activity, where CSCs and non-CSCs are Wnt^hi^ and Wnt^lo^, respectively [11]. Therefore, constant ongoing efforts are being made to identify gene targets that are part of the CSC niche.

Within the dynamic and flexible tumor microenvironment, both matrix-immobilized and soluble proteases are engaged in order to maintain tumor progression [12]. A good example of the robust crosstalk between the proteases in the tumor microenvironment and surface receptors on tumor cells is the family of protease-activated receptors (PARs). Mammalian PARs are part of the large GPCR family, and consist of four members: PAR_1–4_ [13,14,15]. Whereas the evolving role of PARs in tumor advancement has been acknowledged, their underlying molecular mechanism and their signaling-induced post-translational regulation remain elusive. We have previously shown that either PAR_1_ or PAR_2_ oncogenes are effective inducers of β-catenin stabilization, acting via recruitment of LRP5/6 coreceptors in both the malignant and the physiological invasion of placenta anchorage to the uterus decidua [16,17,18,19,20]. PAR_2_ was attributed a dominant role over PAR_1_ [19,21], while PAR_3_ functions mainly as a coreceptor. PAR_4_ is a receptor for thrombin-induced human platelets along with PAR_1_ [22,23]. *Par4*-deficient mice display a normal phenotype; however, hemostasis is diminished, because platelets from these mice no longer respond to thrombin stimulation [24,25]. The information obtained in mice is dissimilar to that for human platelets, in which thrombin effects are mediated by both PAR_1_ and PAR_4_ [13]. On the other hand, thrombin-dependent cleavage of PAR_4_ is critical for leukocyte recruitment and migration to sites of injury, as well as for inflammation [26,27]. In the heart, inhibition of PAR_4_ decreased leukocyte infiltration and cardiomyocyte apoptosis while improving other cardiac tasks following acute ischemia/reperfusion wounds [28,29]. Additional unexpected and surprising properties denote PAR_4_ as an oncogene [27,28,29,30,31]. A survey of a selected GPCR transcription profile using high-throughput RNA sequencing revealed the expression of 195 GPCRs that were either up- or downregulated during somatic reprogramming to CSC sphere formation [32]. Among the GPCRs that were considerably upregulated in CSC sphere formation were PAR_4_ (*f2rl3*) and PAR_2_ (*f2rl1*). As such, they are considered to be part of the CSC niche compartment.

The Polycomb complex is an important effector linking cancer and stem cells [33,34]. It retains stem cell positions by silencing differentiation-associated genes, and is frequently needed for the accurate and precise determination of the differentiation path [33,34]. The catalytic subunit of Polycomb repressive complex 2 (PRC2) is EZH2, Enhancer of Zest Homologue, which functions by methylating lysine 27 of histone H3 (H3K27me3), which is present on the target genes. This leads further to transcriptional silencing by chromatin condensation [33,34]. EZH2 is often overexpressed in metastatic tumors [35,36]. In breast cancers, EZH2 is associated with the high-grade, ER-negative status of basal-like poor prognosis [36,37,38]. It stimulates cancer cell proliferation, invasiveness, and anchorage-independent growth [36,39,40,41]. Along the same evidential lines, EZH2 inhibition reduces tumor growth rates in vivo [40,41,42,43]. However, the molecular mechanism of EZH2’s non-histone-mediated function in cancer remains poorly understood.

Here, we demonstrate novel post-translational regulation of β-catenin induced by either PAR_2_ or PAR_4_, which subsequently endows β-catenin with enhanced stability. EZH2 is upregulated in PAR-driven tumors as well as in aggressive colon cancer cell lines overexpressing PAR_2_ and PAR_4_. In contrast, no expression of EZH2 was observed in the non-aggressive colon cancer cells, which exhibit very little to nearly null PAR expression. Activation of PAR_2_ or PAR_4_ induces the association of EZH2 with β-catenin and methylation on lysine/s (K) residue/s. Consequently, it prolongs the stabilization of β-catenin and enhances its transcriptional activity. PAR-induced β-catenin contributes centrally to colorectal cancer growth.

## 2. Results

**Figure 1:**
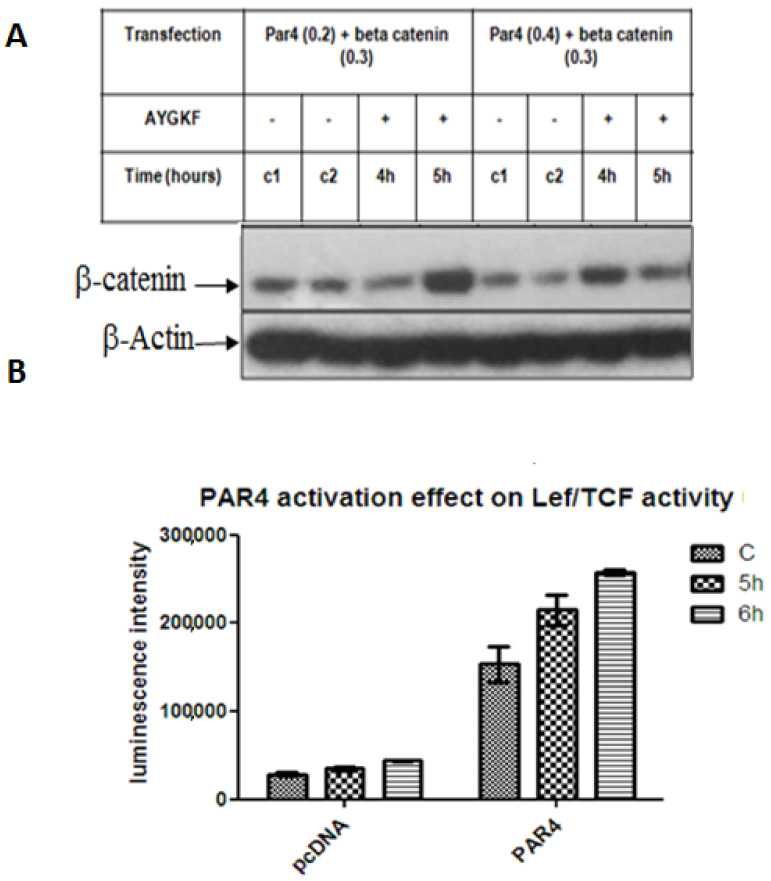
**PAR_4_**-**induced β-catenin stabilization**. (**A**). **β-catenin levels**. HEK293 cells were co-transfected with either 0.2 μg or 0.4 μg *Par4* and 0.3 μg *flag*-β-catenin plasmids and treated with 200 µM AYPGKF for 4 and 5 h to achieve PAR_4_ activation. Western blot analysis was performed on cell lysates to detect β-catenin using anti-*flag* antibody (1:1000) and normalized to β-actin (1:1000) for protein loading. The figure is representative of the assay performed in triplicate. (**B**). **Lef/Tcf tran-scriptional activity**. HEK293 cells were transiently co-transfected with 0.2 μg *Par4*, 0.075 μg TOPflash, 0.125 μg LEF, and 0.2 μg β-gal plasmids. Luciferase activity was normalized to β-gal for transfection efficiency. The mean of duplicates is shown for each treatment. The results are representative of experiments performed in triplicate.

**PAR_4_ induces β-catenin stabilization**. Previously, we have demonstrated the stabilization and TOP*flash* transcriptional activity of β-catenin induced either by PAR_1_ or PAR_2_ [16,17]. Due to the unexpected oncogenic function of PAR_4_, we set out to study the PAR_4_-induced β-catenin stabilization path. For this purpose, HEK293 cells were transiently transfected with *flg*-β-catenin and *Par4*, followed by the addition of AYPGKF, a synthetic hexapeptide exhibiting the internal PAR_4_ ligand sequence for activation. A marked increase in β-catenin levels was observed after 4–5 h of PAR_4_ activation (Figure 1A). The transcriptional activity of β-catenin induced by PAR_4_ was evaluated by the TOPflash luciferase assay carried out in HEK293 cells. Cell lysates were used in the Lef/Tcf Luciferase assay following the transient transfection of *Par4, flg*-β-catenin, β-gal and *lef* plasmids. This demonstrated the increased transcriptional function of β-catenin driven by PAR_4_ (Figure 1B).

**Figure 2:**
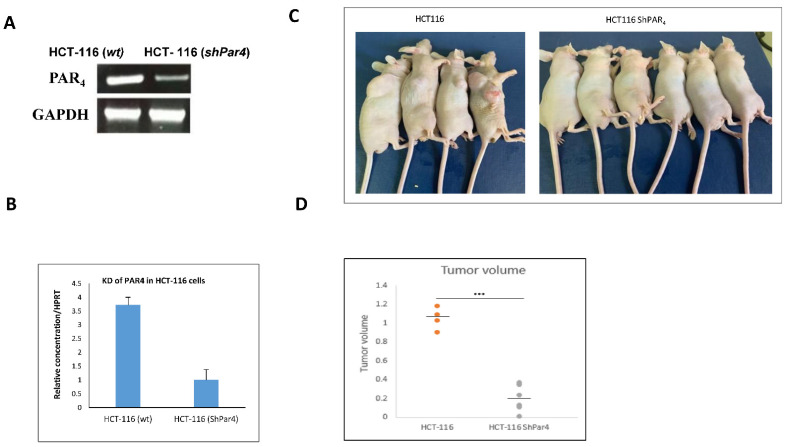
**Knock down of *Par4 i*n HCT116 cells**. (**A**,**B**) **Levels of *Par4* mRNA**. HCT116 cells were stably infected with *sh*RNA-*Par4*. *Par4* mRNA expression was assessed by RT-PCR, normalized to GAPDH (**A**) and by real-time PCR normalized to HPRT (**B**). (**C**,**D**) **Tumor generation in *wt* and *sh*RNA-*Par4* HCT116 cells**. Six-week-old male nude mice were injected subcutaneously with either *wt* HCT116 cells or *sh*RNA-*Par4* HCT116 cells (1 × 10^6^). The experiment was terminated after 5 weeks. These results are representative of the experiment performed three times. Number of mice per each treatment; *n* = 6 (2 died in the HCT116 *wt*-inoculated cells). Tumor volume (**D**) was measured as 0.5 (length) × width^2^.

**Figure 3:**
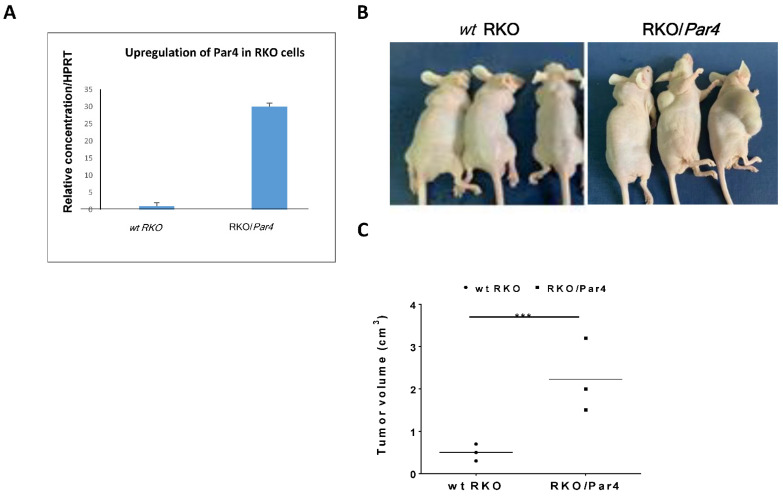
**Tumor generation by RKO/*Par4* clones in nude mice.** (**A**) **Expression of *Par4* mRNA in RKO and RKO/*Par4* clone**. RNA was isolated from the parental RKO cells and clones of RKO/*Par4* (clones; a, b, c). Here, a representative of the RKO/*Par4* clone is shown. Levels of *Par4* were assessed by means of real-time qPCR normalized to HPRT. (**B**) Six-week-old male nude mice were injected subcutaneously with either *wt* RKO cells or *RKO/Par4* clone cells (1.5 × 10^6^). The experiment was terminated after 5 weeks. These results are representative of the experiment performed with two repeititons. Number of mice /treatment *n* = 6 (3 RKO/*Par4* died). RKO/*Par4a* (shown) and RKO/*Par4b* clones were inoculated. (**C**) Tumor volume was measured as 0.5 (length) × width^2^. These results are representative of the experiment performed three times in mice.

**Knock down of *Par4* reduces the otherwise large *Par4*-driven tumors in mice**. The role of *Par4* in tumor generation was further examined by preparing a construct of *sh*RNA-*Par4*, for its silencing of aggressive colon cancer cell lines, while on the other hand, establishing *Par4* clones resulted in the overexpression of the gene in parental RKO cells. HCT-116 cells were stably infected with the lentiviral construct of *sh*RNA-*Par4*. The effectiveness of *sh*RNA-*Par4* silencing in HCT-116 cells following infection with viral particles was evaluated by means of RT-PCR and quantitated by real-time qPCR analyses (Figure 2A,B). Infection of *sh*RNA-*Par4* significantly reduced levels of *Par4* mRNA compared to the non-treated wild-type (*wt*) HCT-116 cells (Figure 2C,D). When nude mice were inoculated with *Par4-*silenced cells, no tumors developed. In contrast, large tumors were generated following inoculation with *wt* HCT-116 cells. This outcome indicates the central role played by *Par4* in the HCT-116 aggressive tumor cell line. To demonstrate the direct impact of PAR_4_ in tumor growth, we generated stable clones expressing *Par4* in RKO cells, a colorectal cancer cell line transformed on the background of mismatch repair system (e.g., of intact β-catenin pathway). These clones were named RKO/*Par4*a–c. The levels of *Par4* mRNA in these cells are shown for a representative clone, RKO/*Par4a* (Figure 3A). When these clones were subcutaneously injected into nude mice (*s.c.*), large tumors were generated compared to the mice injected with parental RKO cells lacking *Par4* expression (Figure 3B,C).

**Figure 4:**
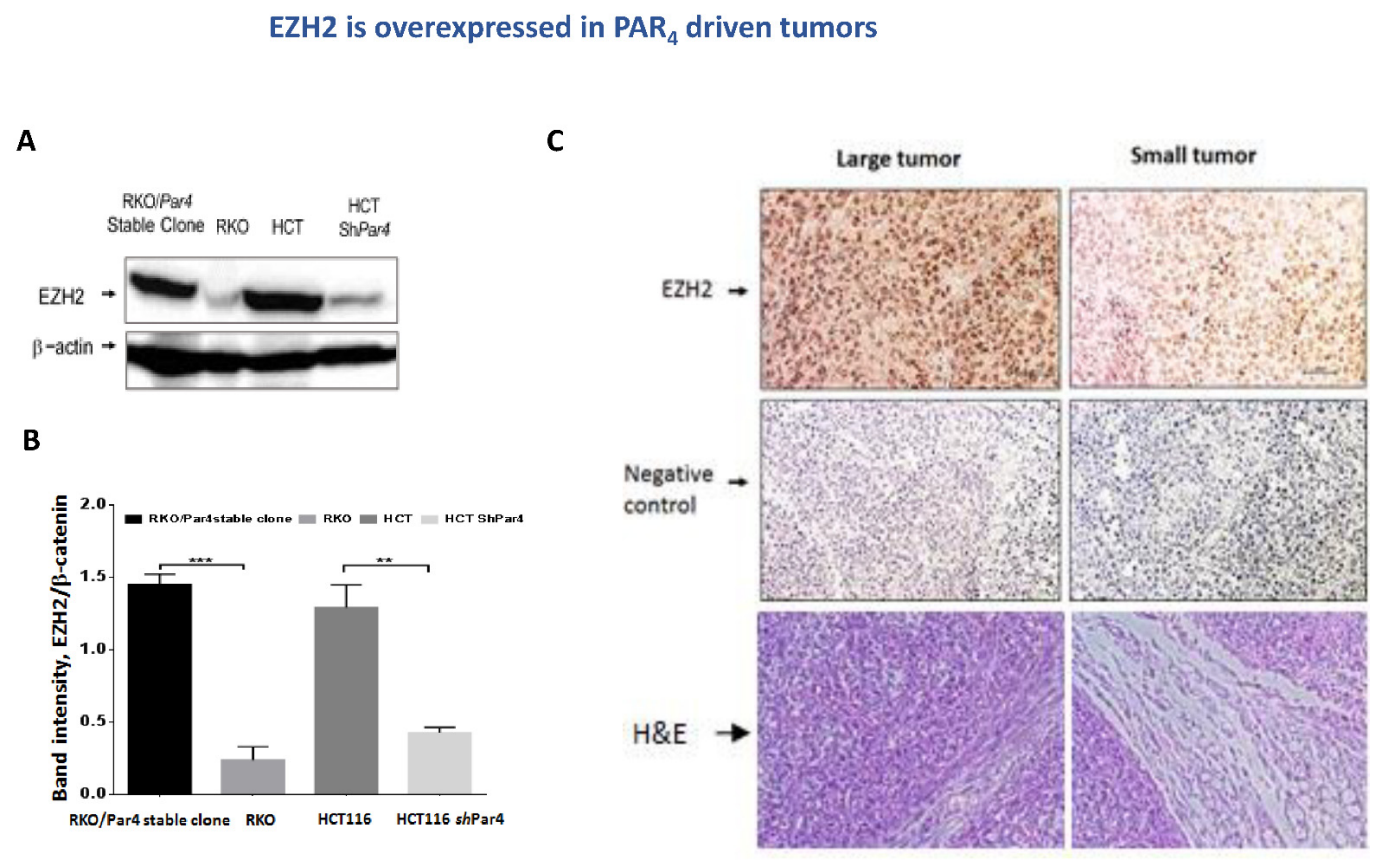
**EZH2 is overexpressed in PAR_4_-driven tumors**. (**A**) Proteins were extracted from tumor lysates: *wt* RKO, *RKO/Par4* clone, *wt* HCT-116, and *sh*RNA-*Par4*-HCT116. Western blot analysis of the lysates was carried out. EZH2 was detected by anti-EZH2 antibodies (1:500) and β-actin by anti-β-actin antibodies (1:1000) as a control for protein loading. Western blot results that are representative of the assays performed three times are shown. (**B**) Quantification of bands using Image J software, normalized to β-Actin. (**C**) **IHC of EZH2 in PAR_4_-driven tumors**. Representative sections of mouse-generated PAR_4_-driven tumors. IHC staining, using anti-EZH2 (1:50 dilution) antibodies. All images were acquired using a Nikon light microscope at magnifications of 10× and 20×. Scale bars 50 µm. EZH2 is abundantly expressed in the large tumors (of high PAR_4_ (RKO*/Par4a* cells)). Very little to almost no EZH2 was detected in the small-appearing tumors (e.g., RKO). As controls for the IHC staining, tissue sections were processed in a similar fashion, but without primary antibodies.

**EZH2 is overexpressed in PAR-driven tumors**. Next, we evaluated the levels of EZH2 in PAR-driven tumors. Western blot analyses of proteins extracted either from tumors generated by RKO/*Par4* clones compared with RKO parental cells, or tumors generated by HCT116 aggressive colon cancer cells versus *sh*-silenced *Par4* HCT116 cells were performed. Pronounced high EZH2 levels were observed in HCT116 as well as in the RKO/*Par4*-generated tumors. In contrast, no expression of EZH2 was observed in tumors generated by *sh*RNA-*Par4* HCT116 cells, nor in RKO non-aggressive cells (Figure 4A,B). Immunohistological (IHC) staining of EZH2 in sections of RKO/*Par4a*-derived tumor tissues compared with tissues of small tumors derived by parental RKO cells resulted in the following outcome. High levels of EZH2 were observed in the tumor section derived from RKO/*Par4a* clone inoculation, while there was nearly none in the tissue sections obtained following inoculation with the RKO parental cells (Figure 4C).

**Figure 5:**
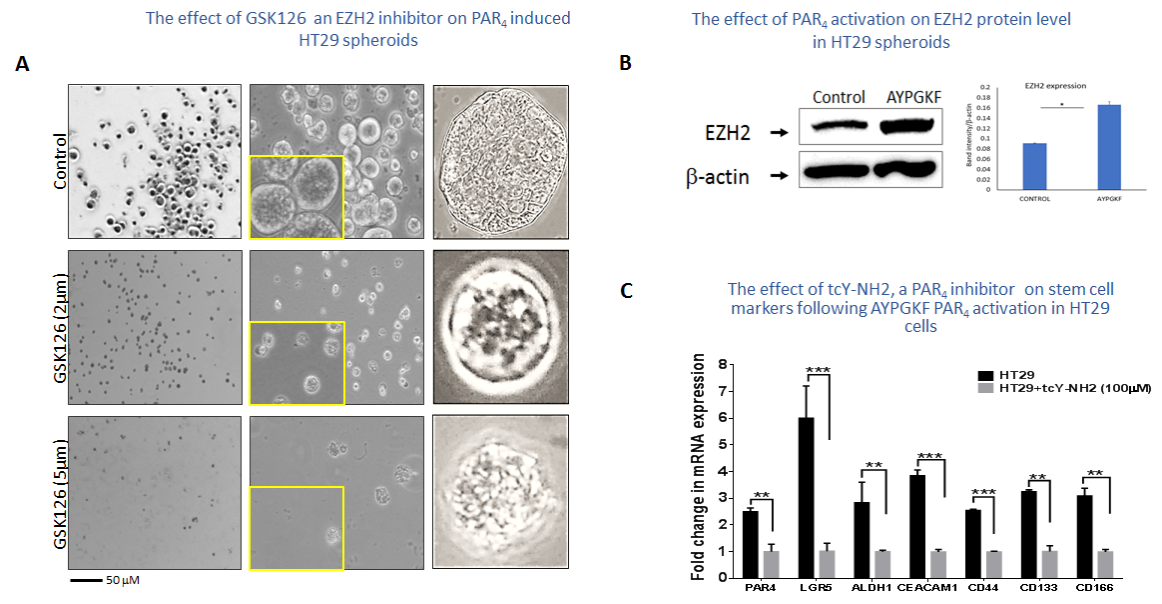
**The effect of GSK126 inhibitor on HT29 spheroids**. (**A**) HT29 cells were grown in a Matrigel layer, supplemented with spheroid formation media. Small spheroids are seen in the presence of 2 μM and 52 μM GSK126, as compared with the *wt* HT-29-generated spheroids. Representative results of the experiment conducted four times are shown. (**B**) **EZH2 level is increased following PAR_4_ activation**. Western blot analyses of protein-lysates prepared from HT-29 spheroids before and after AYPGKF PAR_4_ activation. Increased levels of EZH2 are obtained, as per the β-Actin levels. (**C**) **The effect of tcY-NH_2_, a PAR_4_ inhibitor in HT29 cells, on stem cell markers following AYPGKF PAR_4_ activation**. qPCR analyses of a panel of stem cell markers in the presence and absence of the PAR_4_ inhibitor tcY-NH_2_ (100 μM). An upregulation was observed following AYPGKF PAR_4_ activation, inhibited in the presence of tcY-NH_2_.

**Inhibition of EZH2 by GSK126 inhibits HT-29 spheroid formation**. Spheroid growth and maintenance indicate better cell–cell interactions in vivo [44]. Thus, HT29 cells were grown in the presence of Matrigel and appropriate medium supplements for spheroid formation. When we subjected the spheroids to GSK126, an inhibitor of EZH2 (e.g., 2 μM and 5 μM), the spheres started to undergo apoptosis (Figure 5A). Next, we extracted proteins from the HT-29 spheroids before and after AYPGKF PAR_4_ activation. Western blot analyses showed an increase in the level of EZH2 upon long-term activation of PAR_4_ (Figure 5B). This indicates that activation of PAR_4_ induces EZH2 levels towards a potent association with PAR_4_-induced β-catenin.

**Stem cell markers by PAR_4_**. To further appreciate the significance of PAR_4_ in stimulating stem cell marker expression levels, HT-29 colon cancer cells were subjected to AYPGKF for PAR_4_ activation and either treated or not with tcY-NH2, a potent antagonist of PAR_4_. Next, mRNA was collected, and real-time qPCR was carried out for a panel of stem cell markers known to be elevated by PAR_4_. As can be seen (Figure 5C), all genes analyzed were significantly upregulated by AYPGKF PAR_4_ activation, especially LGR5, as well as CD44 and OCT4. In contrast, spheroids that were activated for PAR_4_ and then treated with tcY-NH2 showed very low expression levels, which is similar to the effect obtained in *sh*RNA-silenced *Par4* (data not shown). Overall, this points to the powerful role played by PAR_4_ as a member of the colon cancer stem cell compartment.

**Figure 6:**
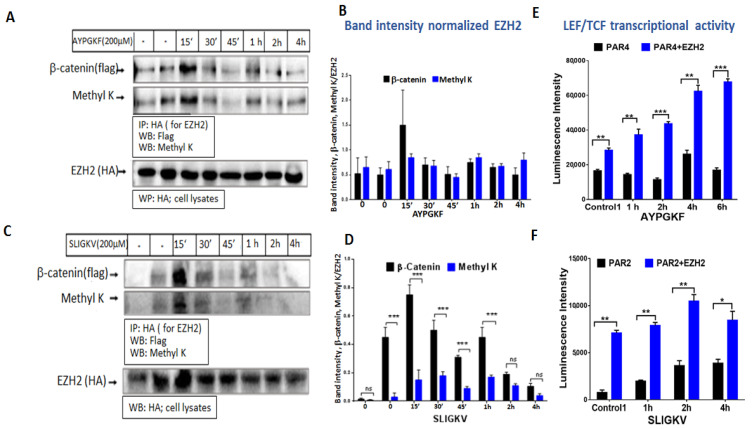
**PAR_4_** (**A**) **and PAR_2_** (**C**) **induced EZH2 binding to β-catenin and lysine (K) methylation.** (**A**,**B**) HEK293 cells were co-transfected with 1.2 μg *Par4* (**A**) or *Par2* (**B**), 2.4 μg HA-*ezh2* and 1.5 μg *flag*-β-catenin. Cell lysates were immunoprecipitated (600 mg per assay) following 200 μM AYPGKF activation (**A**) or 200 μM SLIGKV activation (**B**) for between 15 min and 4 h using anti-HA antibody (3 mL antibody per assay). Detection by Western blot analysis of β-catenin-EZH2 association was performed using anti-*flag* antibody (1:1000 dilution) for β-catenin. Methylation was detected using anti-Methyl K antibody (1:500 dilution). EZH2 levels were evaluated in total cell lysate for normalization (1:500 dilution). Each experiment was performed twice. (**B**,**D**) Quantification of bands was carried out using Image J software. (**E**) **The effect of EZH2 on PAR-activated LEF/TCF transcriptional activity**. (**A**) **PAR_4_ induced Lef/Tcf in the presence of EZH2**. HEK293 cells were co-transfected with 0.075 μg TOPflash, 0.125 μg LEF, 0.2 μg β-gal, 0.2 μg *Par4* and 0.1 μg *ezh2*-HA. Following activation with 200 μM AYPGKF, lysates were collected, and luciferase activity was normalized to β-gal activity to control for transfection efficiency. (**F**) **PAR_2_ induced Lef/Tcf in the presence of EZH2**. HEK293 cells were co-transfected with 0.075 μg TOPflah, 0.125 μg LEF, 0.2 μg β-gal, 0.3 μg *Par2* and 0.3 μg *ezh2*-HA. Following activation with 200μM SLIGKV, lysates were collected, and luciferase activity was normalized to β-gal activity to control for transfection efficiency. These results are representative of the experiment performed in quadruplicate. The results are the mean of duplicates in each experiment. In all our Lef/Tcf experiments, we compared two groups of equally loaded plasmids. The total load of the plasmids did not exceed ~1.1 mg.

**Activation of PAR_4_ or PAR_2_ promotes the binding of EZH2 to β-catenin**. We next examined whether EZH2 interacts with β-catenin following the activation of PAR_4_ or PAR_2_. To this end, immunoprecipitation (IP) analysis between EZH2 and β-catenin was carried out following application of either SLIGKV (for PAR_2_ activation) or AYPGKF (for PAR_4_). Briefly, HEK293 cells were transfected with *flg*-β-catenin, *ezh2* and *Par4* plasmids. Cells were activated for the indicated periods of time, which were between 15 min and 4 h. High levels of β-catenin were obtained within the immune complex of EZH2 following 15 min of PAR_4_ (or PAR_2_) activation, which subsequently decreased. It was concluded that the EZH2–β-catenin association takes place early on, prior to β-catenin stabilization. Concomitantly, lysine (K) methylation of β-catenin was obtained at the same immune complex (Figure 6A,B). EZH2–β-catenin interaction points to a regulatory type of interaction. Notably, similar results were observed following SLIGKV PAR_2_ activation, showing methylation of the β-catenin lysine and association of EZH2 with β-catenin (Figure 6C,D). These data are in agreement with results obtained by Zhu P et al. [9] showing that Lnc-β-Catm associates with β-catenin and EZH2, as can be observed with the AYPGKF activation of PAR_4_ or the SLIGKV activation of PAR_2_.

**Figure 7:**
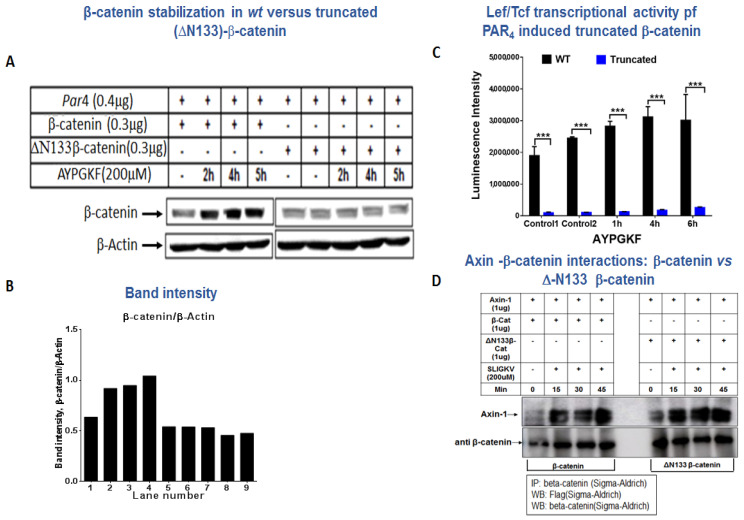
**β-catenin stabilization of *wt* versus truncated (DN133)-β-catenin.** (**A**) Western blot analysis of β-catenin in HEK293 cells. HEK293 cells were co-transfected with 0.4 μg *hPar4* and 0.3 μg *flag*-β-catenin or DN133-β-catenin plasmids and activated by PAR_4_ with 200 µM AYPGKF for 2–5 h. Western blot analysis was performed for cell lysates to detect β-catenin using anti-β-catenin antibody (1:4000) and normalized to β-actin (1:1000) for protein loading. These results are representative of the experiment performed in triplicate. (**B**) Band intensity. (**C**) Lef/Tcf transcriptional activity of *wt* β-catenin and N133 β-catenin. The transcriptional activity was evaluated on the basis of AYPGKF PAR_4_ activity. (**D**) **Association between Axin and either *wt* β-catenin or N133 β-catenin**. HEK293 cells were transfected with *axin*-1, and either *wt* β-catenin or N133 β-catenin. Pull-down immunoprecipitation was carried out using anti-β-catenin antibodies (A5441; Sigma-Aldrich, St. Louis, MO, USA) and WB detection by anti-*flg* antibodies for Axin (F3165; Sigma-Aldrich, USA).

On the basis of the results indicating that activation of PAR_2_ or PAR_4_ induces the association of EZH2 with β- catenin, methylating it on a lysine residue, we next evaluated the effect of this methylation on β-catenin transcriptional activity. To this end, Lef/Tcf luciferase assay was performed in HEK293 cells that were transfected with either *Par4* alone or with both *Par*4 and *ezh2*, as also with *flg*-β-catenin, β-gal and *lef* plasmids. EZH2 expression in the cells resulted in higher levels of Lef/Tcf transcriptional activity following PAR_4_ activation compared to cells transfected with *Par4* alone. Cells transfected with both *ezh2* and *Par4* continued to show markedly enhanced levels of Lef/Tcf activity. These cells exhibited a higher baseline level of Lef/Tcf activity in control non-activated cells (Figure 7A). While the transcriptional activity induced by PAR_4_ alone began to diminish in a timeframe of 6 h following PAR_4_ activation, in the presence of EZH2, Lef/Tcf activity continued to increase (Figure 6E). Similar observations were obtained when SLIGKV activation of cells transfected with *Par2* alone or both *Par2* and *ezh2* was performed. Increased levels were seen after 2 and 4 h of SLIGKV activation, which then subsequently diminished (Figure 6F).

**TruncatedΔN133β-catenin is impaired in β-catenin stabilization**. Three lysine residues are located in the N-terminal portion of β-catenin: K19, K49 and K133. To further evaluate the impact of EZH2 methylation on β-catenin function, a truncated form of β-catenin devoid of 133 N-terminal amino acids, including its lysine residues, was prepared. The truncated β-catenin plasmid—ΔN133β-catenin—was then used to analyze the levels of β-catenin following AYPGKF PAR_4_ activation. While a distinct increase in β-catenin was seen following PAR_4_ activation, no increase was obtained using the truncated form N133β-catenin. Similar data were obtained following PAR_2_ SLIGKV activation of *wt* β-catenin versus truncated ΔN133β-catenin forms (data not shown). This is indicative of the important role of EZH2 in mediating β-catenin methylation followed by PAR_4_ (or PAR_2_)-induced stability of β-catenin (Figure 7A,B).

Transcriptional activity of the truncated ΔN133β-catenin construct was evaluated by means of the Lef/Tcf luciferase assay. HEK293 cells were transfected with *Par4* and *wt* β-catenin or with truncated β-catenin—the N133β-catenin construct—and analyzed for transcriptional activity following AYPGKF PAR_4_ activation. While the *wt* β-catenin plasmid induces abundant and high Lef/Tcf transcriptional activity, cells transfected with the truncated N133β-catenin construct showed a very low level of transcription (Figure 7C). To evaluate the functionality of Δ133β-catenin, we assessed Axin–β-catenin interactions following PAR_2_ SLIGKV activation. This is based on a publication by Li et al. [45] that indicated that Axin binds to β-catenin following Wnt activation. When we analyzed immuno complex formation between Axin and either *wt* β-catenin or Δ133β-catenin, we observed that the 133β-catenin is associated with Axin in a similar manner to *wt* β-catenin (Figure 7D). We therefore conclude that Δ133β-catenin, while devoid of its N-terminal portion, is functional and capable of associating with Axin as part of β-catenin signaling.

**Figure 8:**
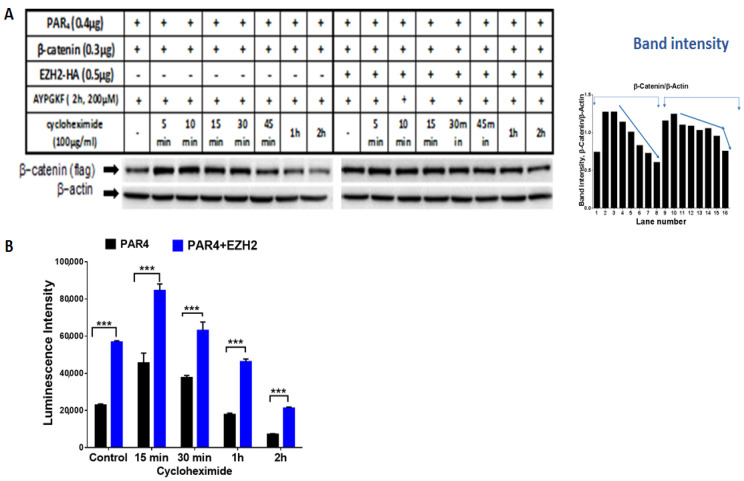
**The effect of EZH2 on PAR_4_-activated β-catenin half-life**. (**A**) Western blot analysis of β-catenin in HEK293 cells co-transfected with 0.4 μg *hPar4*, 0.3 μg *flag*-β-catenin and 0.5 μg *ezh2*-HA plasmids and activated by PAR_4_ with 200 µM AYPGKF for 2 h followed by cycloheximide treatment (100 μg/mL) for between 5 min and 2 h. Western blot analysis was performed on cell lysates to detect β-catenin using anti-*flag* antibody (1:1000) and normalized to β-actin (1:1000) for protein loading. (**B**) HEK293 cells were co-transfected with 0.075 μg TOPflash 0.125 μg LEF, 0.2 μg β-gal, 0.2 μg *Par4*, and 0.1 μg *ezh2*-HA. Following activation with 200 μM AYPGKF and treatment with cycloheximide, lysates were collected, and luciferase activity was normalized to β-gal activity to control for transfection efficiency. This experiment was carried out in triplicate.

**Methylation by EZH2 prolongs β-catenin half-life in PAR_4_ activated cells**. One option to explain the distinct increase in β-catenin levels and transcriptional activity in the presence of EZH2 is the prolonged half-life of β-catenin. To further establish the impact of EZH2 on β-catenin, we analyzed the half-life of β-catenin in the presence and absence of EZH2. This was achieved by using the cycloheximide (CHX) pulse-chase assay. CHX is a known inhibitor of eukaryotic protein synthesis. In the presence of CHX, levels of unstable proteins will decrease, whereas relatively stable proteins will show little change over time [46]. HEK293 cells were transfected with either *β-catenin* and *Par4* plasmids alone, or in combination with *ezh2*. Next, cells were activated with AYPGKF for PAR_4_ activation to stabilize β-catenin, then CHX was added so that no additional proteins could be synthesized. In the presence of *ezh2*, β-catenin expression levels can be seen for longer and sustained periods of time compared to cells transfected with β-catenin and *Par4* alone (Figure 8A,B). This result suggests that EZH2-mediated lysine methylation contributes to the stability of β-catenin, keeping it from being degraded. It is also supported by the prolonged Lef/Tcf transcriptional activity in the presence of EZH2 following the addition of CHX (Figure 8C).

**Scheme 1:**
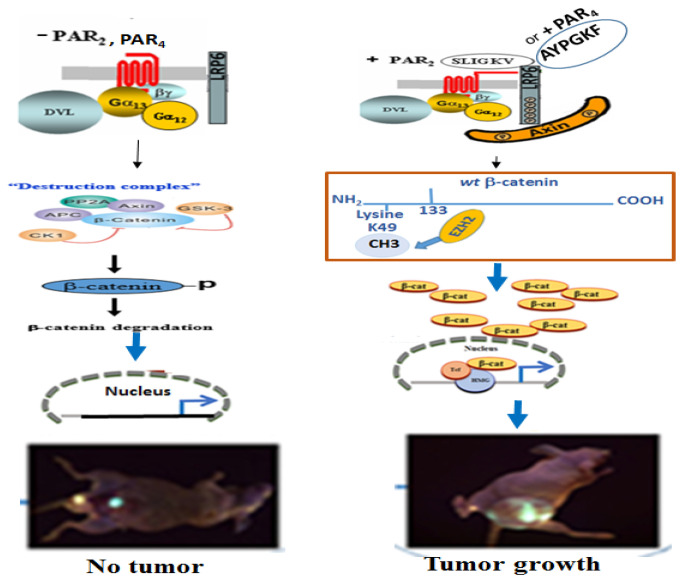
Depicting PAR_2_ or PAR_4_ induced EZH2 association with β-catenin.

**In summary. Induced EZH2 association with β-catenin and methylation on K49**. The activation of either of the PARs leads to the association of EZH2 with β-catenin and methylation of β-catenin on lysine (K) 49. Consequently β-catenin is stabilized and enters the nuclei, where it acts as a co-transcription factor and generates tumors (Scheme 1).

## 3. Discussion

Here we present original data demonstrating the importance of EZH2 non-histone methylation of β-catenin, instigated by PARs. Lysine methylation of β-catenin is essential, and is required for the prolonged stabilization of β-catenin. It unravels an exclusive mode of post-translational regulation of a pivotal player in colon cancer. Supporting data were obtained from the deletion construct of β-catenin devoid of its 133 N-terminal amino acids, lacking, among other things, three lysine (K) residues: K19, K49 and K133. The truncated form of β-catenin exhibits low levels of expression and no transcriptional activity. It is proposed that, early on, methylation of β-catenin is a prerequisite for the stabilization and transcriptional activity of β-catenin. PAR-induced β-catenin stabilization plays a central role in colon cancer growth.

Truncated N133 β-catenin, in addition to lysine amino acids, is devoid of serine/threonine (S/T) residues for phosphorylation. The phosphorylation of β-catenin (on four S/T residues: S33, S37, T41 and S45) assigns it to proteasomal degradation [8,47]. Specifically, CK1α-induced Ser45 phosphorylation generates a priming site for GSK3β, which is necessary for GSK3β-mediated phosphorylation of the Thr41, Ser37, and Ser33 residues. Ser33 and Ser37 in β-catenin form docking sites for the E3 ubiquitin ligase; the β transducing repeat-containing protein (β-TRCP) that ubiquitinates β-catenin and targets it for proteasomal degradation. On the other hand, methylated lysine contributes to the prolonged half-life period for enhanced β-catenin stabilization. Once lysine methylation takes place, it inhibits the tagging of β-catenin by phosphorylation for ubiquitination [9].

Zhu P et al. discovered a long non-coding RNA called lnc-β-Catm (lncRNA for β-catenin methylation) that associates with β-catenin and EZH2, thereby promoting the methylation of β-catenin on lysine 49 (K49) by EZH2. This methylation inhibits β-catenin ubiquitination by the E3 ligase and promotes its stability, thus allowing β-catenin to activate Wnt-β-catenin signaling for a longer period of time, contributing to the sustained stemness of liver CSCs [9]. Our data indicate that the activation of PARs induces the methylation of β-catenin through the association of EZH2, resulting in a longer and continued stability of β-catenin. Whether PAR-induced EZH2 association leads to methylation on K49 alone or K19 in addition still remains to be established.

EZH2 also functions in a PCR2-independent manner. Emerging research has shown that EZH2 methylates non-histone targets interacting with other proteins to activate downstream genes. For example, Kim E et al. discovered that EZH2 binds to and methylates STAT3, leading to its enhanced activity through the increased tyrosine phosphorylation of STAT3 [48]. Another study showed that the phosphorylation of EZH2, mediated directly or indirectly by PI3K/AKT pathway, can switch its function from a Polycomb repressor to a transcriptional co-activator of androgen receptors, and potentially other factors as well [33,35,49].

Mutations of EZH2 are found in hematological malignancies [50]. They promote cancer cell proliferation, anchorage-independent growth, and invasiveness [39,40,41]. In vivo, the inhibition of EZH2 reduces tumor growth rates to various extents [42,43]. Focusing on breast cancer, EZH2 was found to regulate the structure of basal-like cell populations by inducing a ‘bi-lineage’ differentiation state. In this state, cells express both basal and luminal lineage markers [51]. In contrast, GATA3, a driver of luminal differentiation, was demonstrated to carry out a function opposite to that of EZH2, acting to diminish the bi-lineage identity and luminal progenitor gene expression [51].

In fact, EZH2 has been shown to be overexpressed in many cancers, including hepatocellular carcinoma, breast [40], bladder [52], and lung cancer [53]. In prostate cancer, EZH2 has also been used as a molecular marker for poor prognosis [35]. Overexpression of EZH2 is critical for the function of stem cell self-renewal. A mechanism has been described in which EZH2 expression-mediated downregulation of DNA damage repair leads to accumulation of recurrent RAF1 gene amplification in Cancer Initiated Cells (CIC), which activates p-ERK-β-catenin signaling and enhanced CIC growth [54]. As such, a clinical trial (e.g., AZD6244) for a drug that inhibits RAF1-ERK signaling resulted in the inhibition of the progression of breast cancer through the eradication of CICs [50]. In addition, there is a role for EZH2 in immunotherapy. It has been shown to be negatively correlated with CD8+ cytotoxic T cells in ovarian cancer [55].

Although EZH2 works in a variety of ways, including the canonical pathway of epigenetic transcription [56] and gene upregulation, it can also work as a tumor suppressor [57]. For this reason, a precise understanding of the mode by which this protein works, from a molecular level to a cluster network, is essential for developing a future clinical mode of therapy [58].

EZH2 has been shown to bind to and transactivate β-catenin, leading to increased expression of the target genes c-myc and cyclin D1 [59]. C-myc expression also leads to *ezh2* expression by targeting miRNA [60]. Aberrant Wnt signaling in colorectal cancers leads to deregulation of the c-myc gene [61]. Therefore, one could assume that PAR activation inducing Lef/Tcf gene transcription could bring about c-myc expression, and thus influence EZH2 expression. This might explain why when PAR_4_ cells are highly expressed, higher levels of EZH2 expression are exhibited, as indicated in our data. Along this line of evidence, we also showed that the EZH2 inhibitor GSK126 impairs PAR_4_-induced spheroid growth, as well as the growth of stem cell markers that are otherwise markedly elevated by PAR_4_ activation. Taken together, these results emphasize the central role played by EZH2 in PAR_4_-induced malignancy. Elevated levels of several Lef/Tcf transcription targets, such as *lgr5, cd44* and *oct4*, were observed in these spheroids. The expression of these genes is then quelled by inhibition of EZH2 with GSK126.

Overall, it is proposed herein that activation of the GPCR oncogenes PAR_4_ and PAR_2_ potently induce lysine methylation of β-catenin by EZH2, promoting its sustained stability levels in colon cancer.

## 4. Material and Methods

### 4.1. Animal Models

The animals used in the experiments were treated in accordance with the guidelines of the institution ethics committee (AAALAC standard). Mice (HSD: Athymic nude-Foxn1Nu Nu/Nu mice) were kept under specific pathogen-free (SPF) conditions at the Hadassah Medical Center animal facility unit of the Hebrew University and were regularly screened for standard pathogens. All animal experiments were approved by the animal committee of the Hebrew University (MD-20-15924-5).

### 4.2. Cell and Culture Conditions

HCT-116, HT29, RKO and HEK293 (obtained from the American Type Culture Collection, Manassas, VA, USA) were grown in DMEM, supplemented with 1 mM L-glutamine, 50 µg/mL streptomycin, 50 U/mL penicillin (GIBCO-BRL, Gaithersburg, MD, USA) and 10% fetal calf serum (Biological Industries, Migdal HaEmek, Israel). Cells were maintained in a humidified incubator with 8% CO_2_ at 37 °C. To generate spheroids, HT29 cells were cultured for 10–14 days in spheroid media (DMEM with 20 ng/mL hEGF, 20 ng/mL hFGF, 1× B27, 1% L-Glut, 1% Pen-strep A) with 50% Matrigel matrix (Corning, Corning, NY, USA) at 37 °C under 8% CO_2_ in a humidified incubator. Spheroid growth was microscopically monitored daily, and the incubation medium was replaced every 3 days.

### 4.3. Plasmids and Reagents

pBJ-FLAG-h*Par4* (cat #53231), pCMVHA hEZH2 (cat #24230), pcDNA3-mRFP (cat #13032), pCMV-VSV-G (cat #8454) and pCMV-dR8.2 dvpr (cat #8455) plasmids were purchased from Addgene. Human PAR_2_ (*hPar2*/ *f2rl1*) plasmid was kindly provided by Dr. Morley D. Hollenberg (Faculty of Medicine, University of Calgary, Calgary, AB, Canada). *flg*-β-catenin and *flg*-Axin were kindly provided by Dr. Ben-Neriah (Hebrew University, Jerusalem, Israel). All of the mentioned plasmids were sequenced to confirm the absence of undesirable mutations. Details of the plasmids are available on request.

### 4.4. Cell Transfections and PAR Activation

Cells grown to 70–80% confluency were transfected with 0.5–1 µg amounts of plasmid DNA using PEI transfection reagent (Polysciences, Warrington, PA, USA) according to the manufacturer’s instructions. Cells were collected 48 h after transfection, and protein lysates/RNA were prepared. To activate PAR2, a synthetic peptide “SLIGKV” was employed, while to activate PAR4, a synthetic peptide “AYPGKF” (purchased from GenScript; Piscataway, NJ, USA) was used. To inhibit EZH2 activity, we used between 2 µM and 10 µM of GSK126 (ab269816, Abcam, Cambridge, UK) or 4.6 µM of GSK343.

### 4.5. sh-RNA Construct Preparation and Viral Particle Generation

To prepare sh-RNA of PAR4, the sequence was successfully cloned into the plentilox3.7 (pLL3.7) lentiviral vector following the protocol provided by the website of Addgene (Cat#11795). The target sequence was as follows: sh-Par4# (5′- ATGACAGCACGCCCTCAAT′-3). For the generation of shPar4 viral particles, HEK 293 cells were transfected with a three-plasmid system that included packaging CMVD R8.91, envelope (CMV-VSV-G), and shPar4-pLL3.7, using PEI as a transfection reagent. The medium was replaced with fresh medium 24 h later. On day 3 after transfection, the medium was collected, and the viral particles were concentrated 10-fold by centrifuging for 1 h at 40,000× *g* rpm.

### 4.6. Generation of Truncated β-Catenin Construct

To eliminate the methylation of β-catenin, a truncated construct of the *CTNNB1* gene was prepared that was devoid of 133 a.a at its N-terminus. In brief, *CTNNB1* wild-type plasmid template was amplified with Q5 high-fidelity polymerase (NEB, Ipswich, MA, USA), using forward primer (5′ CATGCAGTTGTAAACTTGATTAA 3′) with a *BamH1* restriction site and reverse primer (5′ CAGGTCAGTATCAAACCAG 3′) with a *Not1* restriction site. The amplified product was purified, digested, and cloned into the pcDNA3-RFP vector between the *BamH1* and *EcoRI* sites. Sequences of the plasmid constructs ΔN133-pcDNA-RFP were confirmed by Sanger sequencing.

### 4.7. Generation of the Par4 Construct and Production of Viral Particles

To prepare stable clones of PAR4, the human (*h*)*Par4* gene was amplified with Q5 high-fidelity polymerase (New England Biolabs; NEB, Ipswich, MA, USA) using forward primer (5′GAATTCGCCGCCACCATG TGGGGGCG ACTGCTCC′3) with an *EcoR1* restriction site (underlined) with Kozak sequences (bold) and the reverse primer (5′ACTAGTTCACTGGAGCAAAGA GGAGTGGG′3) with an *Spe1* restriction site, and cloned into the pLVX- EF1 α-IRES-Puro viral vector. For the generation of viral particles, the same procedure was applied as that described earlier.

### 4.8. Preparation of RKO Stable Clones Expressing Par4

RKO (0.5 × 106) cells were infected with hPar4 10× viral particles along with Polybrene infection reagent. At 72 h post transduction, cells were subjected to puromycin selection (0.5 μg/mL). Cells with puromycin resistance were grown and collected, and were either used to isolate RNA or to perform protein lysate preparation.

### 4.9. Quantitative Real-Time PCR (qRT PCR) and RT PCR

RNA was extracted from cells using GenElute RNA kit (Sigma-Aldrich, USA). To prepare the cDNA, 1 µg of RNA was reverse transcribed using reverse transcriptase (Promega, Madison, WI, USA). qRT PCR was conducted using specific forward and reverse primers for each gene listed in Table 1 (the Hprt gene was used as a housekeeping gene for normalization using gene-specific primers). In qRT PCR, triplicates of the 6 ng cDNA template were used with 500 nM gene-specific primers using 2× PerfeCTa SYBR Green mix (Agentek, Tel Aviv-jaffa, Israel) on an automated rotor gene system RG-3000A (Corbett research, Sydney, Australia). All of the data obtained from three independent qRT-PCR experiments were analyzed using the 2^−ΔΔCt^ method as described in the manufacturer’s instructions, and were expressed as fold change over the indicated controls.

To compare expression of Par4 in HCT116 vs. HCT116 *sh*-*Par4*, RT-PCR was performed. The PCR conditions were an initial denaturation at 98 °C for 5 min, denaturation at 98 °C for 30 s, annealing for 60 s at 60 °C, and extension for 1 min at 72 °C (31 cycles of amplification). The GAPDH gene was used as an internal control.

### 4.10. Cell Lysate Preparation

To prepare protein cell lysate for immunoprecipitation, cells were solubilized in CelLytic^TM^M buffer (Sigma-Aldrich, USA), while for the rest of the protein cell lysate preparation, we used lysis buffer containing 10 mM Tris-HCl (pH 7.4), 150 mM NaCl, 1 mM EDTA, 1% triton X-100. All of the mentioned lysis buffers were supplemented with protease inhibitor cocktail, 1 mM phenylmethylsulfonylfluoride, PMSF, and 1 mM Na-orthovanadate (Sigma, St. Louis, MO, USA) to prevent protein degradation. The protein cell lysate used for IP and BA were incubated at 4 °C for 20 min and then disrupted by sonication. Finally, soluble supernatant was collected after centrifugation at 12,000× *g* rpm for 20 min at 4 °C.

Protein cell lysates were separated on 8–10% SDS-PAGE followed by transfer to Immobilon-P membrane (Millipore, Bedford, MA, USA). Membranes were accordingly blocked and probed with the appropriate primary antibodies. Primary antibodies, including anti-flag (F3165; Sigma-Aldrich, USA), anti-β-actin (A5441; Sigma-Aldrich, USA), anti-KMT6/EZH2 (ab191080, Abcam, Cambridge, UK), anti-Methylated Lysine (ab76118, Abcam), anti-HA (901503; Biolegend, San Diego, CA, USA) and anti-beta Catenin antibodies (ab32572, Abcam), were suspended in 3% BSA in 10 mM Tris-HCl pH 7.5, 100 mM NaCl and 0.1% Tween-20. After washing, blots were incubated with secondary antibodies conjugated to horseradish-peroxidase. Immunoreactive bands were detected by the enhanced chemiluminescence (ECL) reagent (Pierce, Rockford, IL, USA). Protein cell lysates (400–800 μg) were used for immunoprecipitation analysis. Anti-HA (sc-7392; Santa Cruz, CA, USA) was added to the cell lysates and processed as previously described [17].

### 4.11. TOPflash Luciferase Reporter Assay

HEK-293T cells (0.2 × 10^6^) were seeded in 6-well plates and incubated overnight at 37 °C. The cells were transfected with the desired target plasmids (PAR2/PAR4/EZH2/β-catenin wt or ΔN133) along with human Lef-1 TOPflash (Tcf Optimal Promoter + luciferase, T cell factor (Tcf) reporter plasmid containing two sets (the second set in reverse orientation) of three copies of the Tcf binding site upstream of the thymidine kinase (TK) minimal promoter and luciferase open reading frame using PEI transfection reagent (Boehringer-Mannheim). CMV/β-gal plasmid was co-transfected as an internal control for transfection efficiency. After 48 h transfection, the cells were washed and lysed, and then luciferase assay was performed with the Luciferase Reporter System (Cat# E1500; Promega, Heidelberg, Germany) according to the manufacturer’s instructions, and luminescence was detected on a Tecan SparkTM10M multimode microplate detection system (Switzerland).

### 4.12. Cycloheximide Chase Assay

To compare the turnover of β-catenin with or without EZH2, plasmids were transfected into HEK293 cells as described above. Following transfection, cells were pre-treated with AYPGKF (200 μM) for 2 h and then exposed to cycloheximide (100 µg/mL) for 5 min to 2 h. Cells were then harvested at regular intervals, and lysates were prepared as mentioned above.

### 4.13. Ectopic Tumor Xenograft Mouse Model Study

To determine the in vivo tumorigenicity of the PAR4/*f2rl3* gene, an ectopic tumor xenograft mouse study was performed. In brief, RKO/PAR4-RKO stable clones or HCT116/HCT116 *sh*PAR4 cells were starved O/N, and the next day were treated with AYPGKF (200 µM) for 4 h. After washing off the cells, 1 × 10^6^ cells were injected subcutaneously into the right flank of groups (*n* = 6) of six- to eight-week-old Hsd: Athymic Nude-Foxn1nu mice (referred to as nude mice). Tumor volumes were monitored twice a week by caliper measurements of each dimension and calculated using the following formula: V = 4/3 π (length/2) (width/2) (depth/2). Mice were terminated by cervical dislocation under aesthetic conditions when the tumor volumes reached the volume stipulated in the Institutional Animal Committee’s approval or when the animals showed distress, in order to avoid unnecessary suffering.

### 4.14. Spheroid Formation Assay

HT29 cells (1 × 10^3^) were grown in sphere formation medium (DMEM supplemented with 20 ng/mL bFGF, 20 ng/mL EGF, and B27) over a layer of 50% Matrigel. Two weeks later, spheres larger than 100 μm were counted, and photographs were taken. Spheroid lysates were prepared in the same manner as that described above (solubilized in 10 mM Tris-HCl (pH 7.4), 150 mM NaCl, 1 mM EDTA, 1% triton X-100).

### 4.15. Immunohistochemistry

Paraffin-embedded slides derived from PAR4-RKO tumor tissue compared with small tumor tissue derived from parental RKO cells were used for IHC. After deparaffinization and rehydration, the slides were incubated with 3% H2O2 prior to antigen retrieval. Antigen unmasking was carried out by heating (20 min) in a microwave oven with 1× antigen retrieval citrate buffer (Cat# ab93678, Abcam). After blocking with CAS-Block (Cat# 008120, Invitrogen, MA, USA), the slides were incubated with EZH2 antibody (Cat# ab191080; Abcam, Cambridge, UK). Next, following washing, the slides were incubated with peroxidase-conjugated antibody (Abcam, Cambridge, UK). Color was developed using the DAB substrate kit (Cat# 34002, Thermo Scientific, Waltham, MA, USA), followed by counter staining with Mayer’s hematoxylin (Cat# 3801582E, Leica, Wetzlar, Germany). Controls using only secondary antibodies (with no primary antibodies) showed low to background staining in all cases.

### 4.16. Statistical Analyses

All of the experiments were carried out in triplicate, whereby the data are represented as mean ± SD. Significant differences in the tested samples in comparison to control were determined by performing either Student’s *t* test or analysis of variance (ANOVA) with Tukey’s multiple comparison post test (GraphPad Prism 6.0; Bioz Stars, Los Altos, CA, USA), wherever required. The criterion for statistical significance was as follows: *p* < 0.05 was considered significant (*), *p* < 0.01 as highly significant (**), and *p* < 0.001 as very highly significant (***).

## Figures and Tables

**Table 1:** List of specific forward and reverse primers for each gene when qRT PCR was conducted.

Gene	Forward Primer seq.	Reverse Primer seq.
hprt	5′ACTGGCAAAACAATGCAGACTTT′3	5′GGTCCTTTTCACCAGCAAGCT′3
h*Par4*	5′CCCAGCGTCTACGACGAGA′3	5′CACAGACTTGGCCTGGGTAG′3
LGR5	5′CTTCCAACCTCAGCGTCTTC3′	5′TTTCCCGCAAGACGTAACTC3′
CEACAM1	5′GCTTCTGCTCACAGCCTCAC3′	5′CCTTCCCCTCTGCAACATTGA3′
CD44	5′CTGGGGACTCTGCCTCGT3′	5′ACGTGGAATACACCTGCAAAGC3′
ALDH1	5′CACGCCAGACTTACCTGTCC3′	5′TGCCACTCACTGAATCATGCC3′
OCT4	5′AGGAGAAGCTGGAGCAAAACC3′	5′ATCCTCTTCTGCTTCAGGAGCT3′
OLFM1	5′GGCCAAGGTAGTGGTACAGC3′	5′CTTGCCAAGCAACATTAGCA3′
CyclinD1	5′ACAAACAGATCATCCGCAAACAC3′	5′TGTTGGGGCTCCTCAGGTTC3′

## Data Availability

The data that support findings of this study are available at the corresponding author upon request.
